# Catalysis by the Tumor-Suppressor Enzymes PTEN and PTEN-L

**DOI:** 10.1371/journal.pone.0116898

**Published:** 2015-01-21

**Authors:** Sean B. Johnston, Ronald T. Raines

**Affiliations:** 1 Department of Biochemistry, University of Wisconsin-Madison, Madison, Wisconsin, United States of America; 2 Department of Chemistry, University of Wisconsin-Madison, Madison, Wisconsin, United States of America

## Abstract

Phosphatase and tensin homologue deleted from chromosome ten (PTEN) is a lipid phosphatase tumor suppressor that is lost or inactivated in most human tumors. The enzyme catalyzes the hydrolysis of phosphatidylinositol-(3,4,5)-trisphosphate (PIP_3_) to form phosphatidylinositol-(4,5)-bisphosphate (PIP_2_) and inorganic phosphate. Here, we report on the first continuous assay for the catalytic activity of PTEN. Using this assay, we demonstrate that human PTEN is activated by the reaction product PIP_2_, as well as in solutions of low salt concentration. This activation is abrogated in the K13A variant, which has a disruption in a putative binding site for PIP_2_. We also demonstrate that PTEN-L, which derives from alternative translation of the *PTEN* mRNA, is activated constitutively. These findings have implications for catalysis by PTEN in physiological environments and could expedite the development of PTEN-based chemotherapeutic agents.

## Introduction

**Figure 1:**
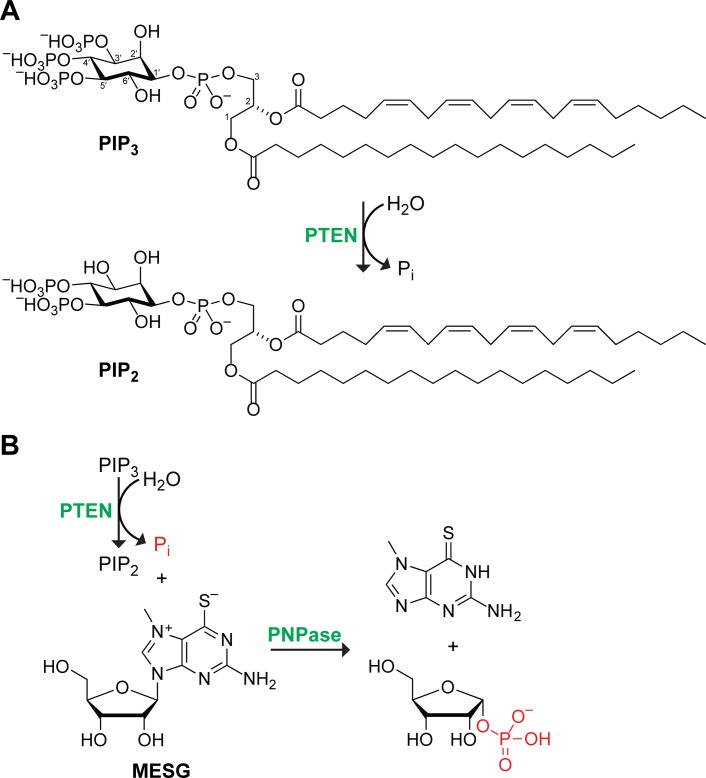
Scheme of assay for catalysis by PTEN. (A) PTEN catalyzes the hydrolysis of phosphatidylinositol-3,4,5-trisphosphate (PIP_3_) to form inorganic phosphate and phosphatidylinositol-4,5-bisphosphate (PIP_2_). Canonically, the fatty acids are arachidonic acid (20:4) and stearic acid (18:0). (B) In the coupled enzyme assay, purine nucleoside phosphorylase (PNPase) catalyzes the reaction of the inorganic phosphate produced by PTEN with 7-methyl-6-thioguanosine (MESG). Upon release, 7-methyl-6-thioguanine undergoes tautomerization, leading to a marked increase in absorbance at 360 nm.

The *PTEN* gene located at human chromosome 10q23 encodes the tumor suppressor enzyme PTEN (EC 3.1.3.67) [1–3]. *PTEN* is thought to be mutated in 50–80% of sporadic human cancers [4]. In the 15 years since the discovery of its canonical substrate, phosphatidylinositol-(3,4,5)-trisphosphate (PIP_3_) [5], PTEN has entered center-stage for cancer biologists. By catalyzing the dephosphorylation of PIP_3_ (Fig. 1A), PTEN antagonizes the phosphatidylinositol-3-kinase (PI3K) Akt pathway, mediating cell proliferation and apoptosis. In addition to its phosphatase activity on PIP_3_, PTEN dephosphorylates protein substrates in the cytosol [6]. PTEN has roles elsewhere in the cell, specifically in the nucleus [7], where it is thought to have effects on gene expression, genomic stability, and cell-cycle regulation [8].

Human PTEN has 403 amino-acid residues (47.2 kDa). The enzyme consists of two globular domains: a phosphatase domain with homology to dual specificity phosphatases and a C2 domain with homology to phospholipase C1 (PLC1) [9]. The phosphatase domain contains a canonical protein tyrosine phosphatase motif, HCXXGXXR [10].

Hypomorphic mouse models have revealed that subtle reductions in the level of PTEN and thus its enzymatic activity can lead to large cancer-related phenotypes [11]. Extant assays to measure the catalytic activity of PTEN are, however, imprecise and tedious. Catalytic activity with canonical phosphatase substrates, such as *p*-nitrophenylphosphate, is too low to be useful [12]. Instead, the activity of PTEN is most often measured with a discontinuous assay based on the formation of complex between the organic dye malachite green and inorganic phosphate [13] produced from a soluble lipidic [14,15] or liposomal [16] PIP_3_ substrate. The assay has a limited range, which ends at ∼20 μM phosphate in a 10-mm cuvette. Still, in assays using soluble lipidic and liposomal substrates, the malachite green-based assay has revealed that PTEN is activated by its PIP_2_ product, which binds near the N terminus.

In 2013, Parsons and coworkers reported on a form of PTEN called PTEN-L (64.9 kDa), which is translated from a conserved CUG codon [17]. The alternative form has 173 additional N-terminal amino-acid residues, including a secretion signal sequence and an Arg_6_ motif that enables extracellular protein to enter the cytosol. When injected into mice lacking PTEN, PTEN–L caused tumors to regress completely. PTEN-α, which is an intracellular version of PTEN-L, is translated from the same CUG codon [18]. PTEN-L has enzymatic activity, but its catalysis is even less well characterized than that of wild-type PTEN.

We are aware that PTEN is a lipid phosphatase—its catalytic activity *in cellulo* is manifested on the inner leaflet of the plasma membrane [1–3]. In an attempt to model catalysis *in cellulo*, assays of PTEN have employed a PIP_3_ substrate embedded on the outer leaflet of a synthetic liposome [16]. These complex assays cannot be used to dissect fundamental aspects of catalysis by PTEN. For example, the multivalency that arises from binding to the PIP_2_ product that is generated on the surface of a liposome enhances the affinity of PTEN for a PIP_3_ substrate on that liposome. Hence, the degree to which PTEN is activated by individual molecules of PIP_2_ is not apparent from data derived from liposome-based assays.

The enzyme purine nucleoside phosphorylase (PNPase; EC 2.4.2.1) catalyzes the reaction of inorganic phosphate with purine nucleosides to displace a purine nucleobase. Tautomerism within the nonnatural purine nucleobase 7-methyl-6-thioguanine leads to a large increase in its absorbance as a nucleobase rather than a nucleoside, and can serve as the basis for a sensitive, continuous assay for inorganic phosphate [19], like that produced by PTEN.

Here, we present the first continuous assay for the catalytic activity of PTEN. Our assay couples inorganic phosphate production by PTEN to catalysis by PNPase (Fig. 1B). Using this assay, we find that the catalytic activity of human PTEN is much greater than reported previously, and that the activity of PTEN-L is comparable to that of PTEN. Finally, using a PTEN variant that lacks the binding motif for the phosphatidylinositol-(4,5)-bisphosphate (PIP_2_) product [20], we delineate dramatic allosterism in catalysis by the wild-type enzyme. These findings underpin an understanding of the biology and pharmacology of PTEN.

## Results

### Production and Purification of PTEN

**Figure 2:**
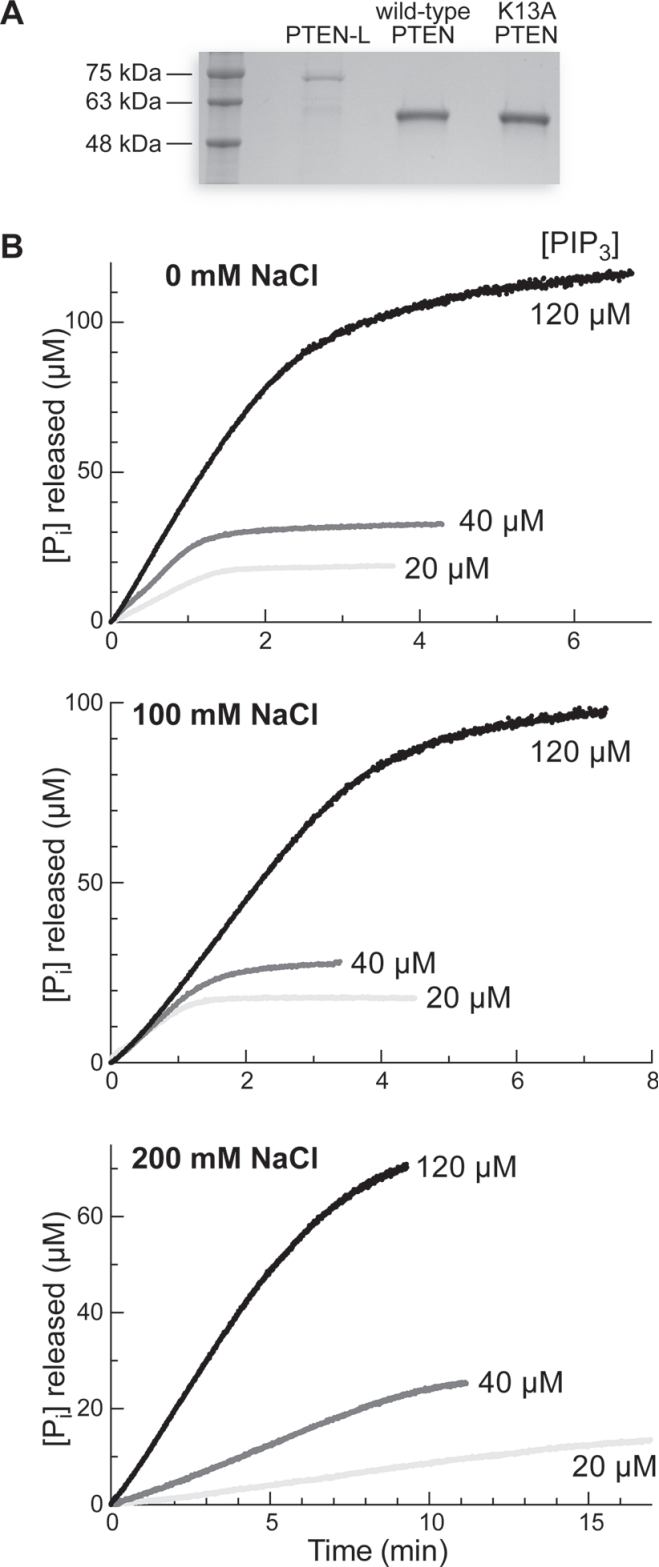
Initial characterization of PTEN. (A) Analysis of PTEN-L, wild-type PTEN, and K13A PTEN on a Coomassie R-250–stained polyacrylamide (12% w/v) gel containing sodium dodecylsulfate (0.1% w/v). (B) Representative progress curves for the turnover of PIP_3_ (10, 15, or 40 μM) by wild-type PTEN. Reactions were performed in 50 mM Tris–HCl buffer, pH 7.6, containing EDTA (2.0 mM), MESG (0.20 mM), DTBA (40 mM), and NaCl (0, 100, or 200 mM), and were initiated with the addition of PTEN to 20 nM. Absorbance at 360 nM was converted to concentration by using Δ*ε* = 11 mM^−1^cm^−1^.

Wild-type PTEN, K13A PTEN, and PTEN-L were produced in *Escherichia coli* by recombinant DNA technology. Each protein had a C-terminal His_6_-tag and was purified by Ni-affinity and gel-filtration chromatography, along with either anion exchange chromatography (wild-type PTEN and its K13A variant) or heparin-affinity chromatography (PTEN-L). Enzyme purity was assessed with SDS–PAGE (Fig. 2A), and typical isolated yields of wild-type PTEN, K13A PTEN, and PTEN-L were 5, 5, and 1 mg per L of *E. coli* culture, respectively.

Earlier this year, Pandolfi and coworkers reported qualitative data suggesting that the overproduction of PTEN can lead to its dimerization *in cellulo* [21]. Yet, a quantitative analysis of the proteome of U2OS osteosarcoma cells growing in log phase detected <500 molecules of PTEN per cell, equivalent to a concentration of <0.2 nM [22]. At this low concentration, even with a nanomolar value of *K*_d_, only a small fraction of PTEN would exist in a dimeric form. When we purify PTEN by gel-filtration chromatography at 100 mM NaCl, we observe only one peak corresponding to monomeric PTEN. (Concentrations of PTEN in these purifications approach 50 μM.) As our assays are performed at enzyme concentrations of ≤250 nM, we believe that our data report on catalysis by monomeric PTEN, replicating the state *in cellulo*.

### Catalysis by PTEN in the Absence of Salt Exhibits Michaelis–Menten Kinetics

Representative time courses revealed that rates measured with our coupled assay were dependent on the concentration of the PIP_3_ substrate (Fig. 2B). We observed 85–95% conversion of PIP_3_ to PIP_2_ in all assays. In a proper coupled assay, the observed reaction velocity must be for the target enzyme and its substrate rather than the coupling enzyme and its substrate [23]. We found that doubling the concentration of PNPase or MESG had no effect on observed rates (data not shown), validating our assay.

**Figure 3:**
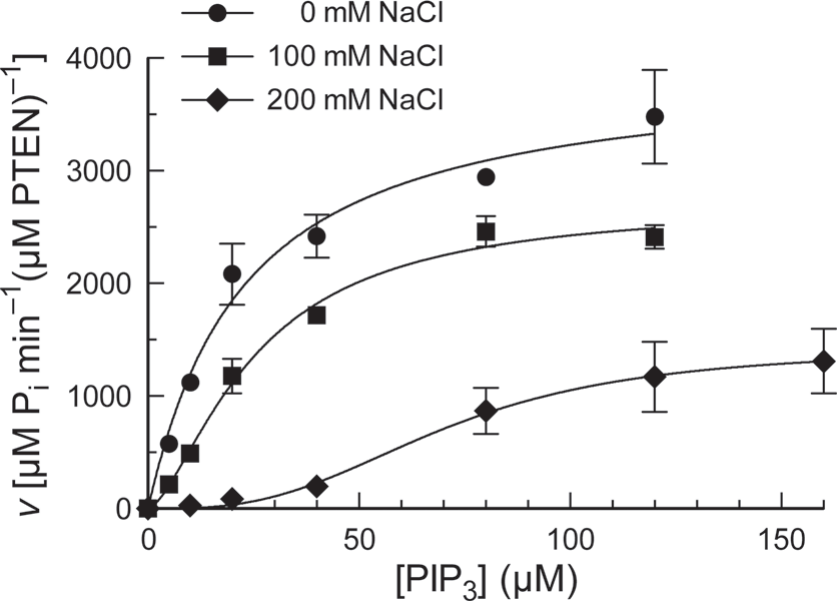
Turnover of PIP_3_ by wild-type PTEN in solutions of various salt concentrations. Reactions were performed in 50 mM Tris–HCl buffer, pH 7.6, containing NaCl (0, 100, or 200 mM), EDTA (2.0 mM), MESG (0.20 mM), and DTBA (40 mM), and were initiated with the addition of PTEN to 20 nM. Values are for maximum reaction velocity (± SE) at <10% turnover of substrate in reactions performed in triplicate or more. The resulting kinetic parameters are listed in Table 1.

Using our continuous assay, we discovered that salt concentration has a marked influence on catalysis by PTEN (Fig. 3). When we refrained from adding salt to our assay solution, we were surprised to discover that PTEN exhibits Michaelis–Menten kinetics. This finding is in marked contrast to results from assays done in the presence of salt [20,24], and the results are especially disparate at low substrate concentrations (<10 μM PIP_3_). By using non-linear regression to fit the initial velocity data to the Michaelis–Menten equation (eq 1):

$$v_{o}=\frac{k_{\mathrm{cat}}\left[E\right]\left[S\right]}{K_{M}+\left[S\right]}$$ (1)

we found that *k*_cat_/*K*_M_ = (174 ± 40) μM^−1^min^−1^ = (2.8 ± 0.6) × 10^6^ M^−1^s^−1^, which is 10^1^- to 10^2^-fold larger than values obtained previously [15,17,20,25].

### Catalysis by PTEN Exhibits Salt-dependent Cooperativity

Previous assays of the enzymatic activity of PTEN and other phosphoinositide phosphatases have been performed in the presence of added salt [20,26–28]. We found that, in the presence of salt at physiological concentrations, PTEN exhibits cooperative activity, which can be described by the Hill equation, adapted for enzymatic catalysis:

$$v_{o}=\frac{k_{\mathrm{cat}}\left[E\right]{\left[S\right]}^{h}}{K_{0.5}^{h}+{\left[S\right]}^{h}}$$ (2)

where *h* is the Hill coefficient [29]. When we performed our coupled assay in the presence of 100 mM and 200 mM NaCl, we saw a drop in enzymatic activity at all substrate concentrations, but a striking drop in activity at low concentrations of substrate (Fig. 3). When fitted with the Hill equation, the data indicated Hill coefficients at 100 and 200 mM NaCl that differed significantly from unity (Table 1). In contrast, we found the Hill coefficient for reactions without salt to be *h* = 1.03 ± 0.33. These data indicate that the extent of cooperativity of catalysis by PTEN is correlated strongly with salt concentration. Although NaCl is the salt used in most assay solutions for PTEN activity, we found that other salts (*e.g.*, KCl, NaNO_3_, and NH_3_Cl) elicit similar effects (data not shown).

**Table 1:** Kinetic parameters for catalysis by wild-type PTEN, K13A PTEN, and PTEN-L*^a^*.

**Enzyme**	**[NaCl] (mM)**	***k*_cat_/*K*_M_ (μM^–1^min^–1^)*^b^***	***k*_cat_ (min^–1^)**	***K*_M_ (μM)**	***h^c^***
Wild-type PTEN	0	174 ± 40	4000 ± 300	23 ± 5	1.00 ± 0.14
	100	108 ± 14***^c^***	2720 ± 130***^c^***	25 ± 3***^c^***	1.54 ± 0.16
	200	20 ± 4***^c^***	1430 ± 200***^c^***	70 ± 11***^c^***	2.93 ± 0.92
K13A PTEN	0	1.4 ± 0.4	91 ± 9	64 ± 16	1.25 ± 0.33
	100	1.0 ± 0.3	101 ± 12	100 ± 25	1.36 ± 0.35
	200	0.39 ± 0.13	139 ± 24	359 ± 96	1.35 ± 0.21
PTEN-L	0	100 ± 25	477 ± 28	4.8 ± 1.1	0.84 ± 0.30
	100	42 ± 8	515 ± 30	12 ± 2	1.23 ± 0.31
	200	10 ± 2	477 ± 40	47 ± 8	1.19 ± 0.17

^a^ Reactions were performed in 50 mM Tris–HCl buffer, pH 7.6, containing NaCl (0, 100, or 200 mM), EDTA (2.0 mM), MESG (0.20 mM), and DTBA (40 mM), and were initiated with the addition of PTEN to 20 nM.

^b^ Values (± SE) were derived by fitting initial velocity data to eq 1, unless noted otherwise.

^c^ Values (± SE) were derived by fitting initial velocity data to eq 2, and include *k*_cat_/*K*_0.5_ and *K*_0.5_ rather than *k*_cat_/*K*_M_ and *K*_M_.

### Catalytic Activity of PTEN is Diminished by Disruption of the PIP_2_-Binding Motif

**Figure 4:**
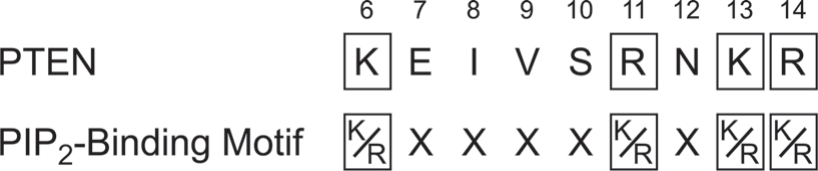
Residues Lys-6 to Arg-14 of PTEN and the canonical PIP_2_-binding motif. Each conserved residue in this motif is altered in cancer according to the COSMIC database.

Next, we explored whether the salt-dependent cooperativity that we observed with PTEN is due to the purported PIP_2_-binding motif of its N terminus (Fig. 4) [30]. Previous studies suggested that disruption of this motif could disrupt activation and lower overall activity [20]. Accordingly, we made a K13A substitution to disrupt this motif.

**Figure 5:**
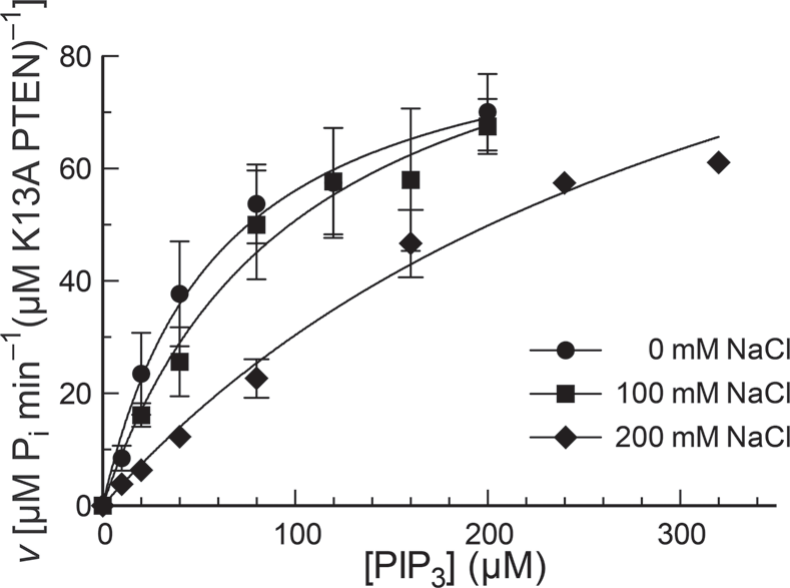
Turnover of PIP_3_ by K13A PTEN in solutions of various salt concentrations. Reactions were performed in 50 mM Tris–HCl buffer, pH 7.6, containing NaCl (0, 100, or 200 mM), EDTA (2.0 mM), MESG (0.20 mM), and DTBA (40 mM), and were initiated with the addition of K13A PTEN to 20 nM. Values are for maximum reaction velocity (± SE) at <10% turnover of substrate in reactions performed in triplicate or more. The resulting kinetic parameters are listed in Table 1.

In the absence of salt, K13A PTEN displays much less catalytic activity than does the wild-type enzyme (Fig. 5). Fitting the data to eq 1 gives a value of *k*_cat_/*K*_M_ = (1.4 ± 0.4) μM^−1^min^−1^, which is ∼1% that of wild-type PTEN (Table 1). At low concentrations of substrate (10 and 20 μM), the velocity of catalysis by K13A PTEN in the absence of salt resembles that of wild-type PTEN in the presence of 200 mM NaCl.

Upon addition of salt, the catalytic activity of K13A PTEN is diminished still further (Fig. 5). All curves, including those in the absence of salt, are slightly sigmoidal, perhaps showing marginal product activation at high substrate concentrations. Fitting to the Hill equation (eq 2) gives Hill coefficients that might differ significantly from unity (Table 1).

### Catalytic Activity of PTEN-L is Constitutive

A form of PTEN is produced from an alternative translation-initiation codon (CUG) upstream on the canonical PTEN mRNA [17]. Alternative translation-initiation adds 173 amino-acid residues to the N terminus of the protein. Due to the location of the additional residues on the N terminus, we hypothesized that the added domain could affect the activating effect of PIP_2_.

**Figure 6:**
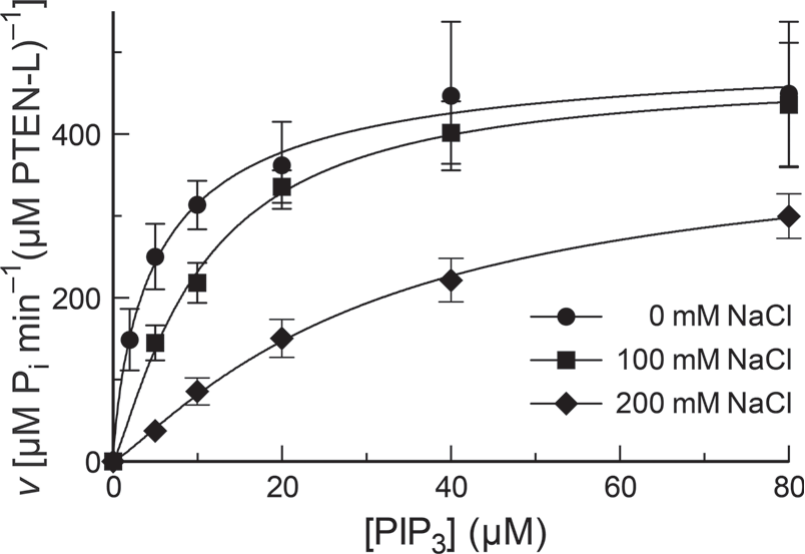
Turnover of PIP_3_ by PTEN-L in solutions of various salt concentrations. Reactions were performed in 50 mM Tris–HCl buffer, pH 7.6, containing NaCl (0, 100, or 200 mM), EDTA (2.0 mM), MESG (0.20 mM), and DTBA (40 mM), and were initiated with the addition of PTEN-L to 20 nM. Values are for maximum reaction velocity (± SE) at <10% turnover of substrate in reactions performed in triplicate or more. The resulting kinetic parameters are listed in Table 1.

Indeed, our data indicate that PTEN-L is 5-fold more effective at binding to its substrate than is wild-type PTEN (Fig. 6) with *K*_M_ = (4.8 ± 1.1). Fitting the data to the Michaelis–Menten equation (eq 1) gives *k*_cat_/*K*_M_ = (100 ± 25) μM^−1^min^−1^ = (1.7 ± 0.4) × 10^6^ M^−1^s^−1^.

Supplementation of salt to physiological levels did not seem to introduce allosterism, evident by the absence of sigmoidal kinetics (Fig. 6). Fitting to the Hill equation (eq 2) gives Hill coefficients that do not differ significantly from unity (Table 1). These data are consistent with the N terminus of PTEN-L replicating the activating effect of PIP_2_ and maintaining an activated state of PTEN.

## Discussion

PTEN is an important phosphatase tumor suppressor with many roles in the cell [8]. Loss of PTEN, whether by mutation or by posttranslational inactivation, is causative in 50–80% of sporadic cancers [4]. PTEN-L is a recently discovered secreted and membrane-permeable translational variant of PTEN [17]. Although many assays of the phosphatase activity of PTEN have been established, none has been continuous. In this work, we establish a continuous assay for PTEN hydrolysis of its endogenous substrate, PIP_3_, reveal new aspects of activation by the product, PIP_2_, confirm the location of the PIP_2_-binding site, and demonstrate that PTEN-L does not require PIP_2_ to retain an activated conformation. Other assays of PTEN activity have employed solutions of diverse salt concentration [14,26], a parameter that we find to have large effects on PTEN activity. In addition, we measure values of *k*_cat_/*K*_M_ that are more than an order-of-magnitude larger than those derived from other assays, indicating that PTEN and PTEN-L are much better catalysts than appreciated previously. Although the high values of *k*_cat_/*K*_M_ are due to the absence of salt, we also employ a highly purified enzyme (Fig. 2A) with a minimal tag (His_6_) and a superior reducing agent (DTBA [31]) to maintain the active-site cysteine residue in its reduced state. This residue is extremely sensitive to oxidation.

In previous work, we studied the effect of salt concentration on the ability of an enzyme to turnover its phosphorylated substrate [32]. In general, we found high salt concentrations to lower the value of *k*_cat_/*K*_M_, but not affect that of *k*_cat_. Salt counterions are associated with the enzyme and substrate prior to binding, and the dissociation of those ions during binding and consequent gain of entropy is determinant in raising the value of *K*_M_. Here, we observe analogous effect on the value of *K*_M_ for PTEN, which increase with increasing salt concentration (Table 1). In contrast, we also observe analogous effects on the value of *h* for the wild-type enzyme, which likewise increases with increasing salt concentration. These data indicate that the binding of both PIP_3_ to the active site and PIP_2_ to the PIP_2_-binding site are mediated by favorable Coulombic interactions. Moreover, the latter interaction appears to be especially strong at low salt concentration, where the value of *h* = 1.03 suggests that wild-type PTEN is readily saturable by PIP_2_.

We are aware of another physicochemical explanation for a decrease in enzymatic activity at high salt concentration. Specifically, the addition of salt could entice a protein to aggregate via the hydrophobic effect, a process known as “salting out” [33,34]. We note, however, that the effect of salt concentration on the catalytic activity of PTEN is is not constant but depends on the concentration of PIP_3_, with the largest impact occurring at low PIP_3_ concentrations (Fig. 3). These data, along with the absence of turbidity in our assay solutions, indicate that PTEN aggregation is not responsible for the decrease in its catalytic activity at high salt concentration.

Catalysis by PTEN has notable elements of cooperativity. In the absence of salt, this cooperativity disappears, PTEN is activated fully, and kinetic data become amenable to Michaelis–Menten analysis. In its cellular environment, however, catalysis by PTEN is cooperative. Activation by PIP_2_ has been noted in assays using soluble lipidic or liposomal substrates [14–16]. Other phosphotidylinositide phosphatases appear to exhibit a similar product-activating phenotype. For example, product activation has appeared in assays of myotubularins (PI(3,5)P_2_→PI(5)P) [26], an insect-derived salivary inositol phosphatase (PI(4,5)P_2_→PI(4)P) [27], and eukaryotic Sac1 (PI(4)P→PI) [28]. Limitations in extant assays have prevented detailed analysis of this kinetic behavior. Aside from phosphatidylinositol phosphatases, product activation like that of PTEN is a rare phenomenon. There are, however, other monomeric enzymes that display cooperativity, due mostly to large conformational changes [35].

Disrupting the PIP_2_-binding motif (Fig. 4) reduces the catalytic activity of PTEN severely (Fig. 5). In addition, this disruption also abrogates the sigmoidal nature of substrate–velocity curves. These data are consistent with a mechanism of cooperativity that involves the product, PIP_2_, binding to its N-terminal motif and activating the enzyme for catalysis of PIP_3_ hydrolysis [20]. This kinetic mechanism ensures that PTEN is most active at membranes, which present both the PIP_3_ substrate and PIP_2_ product. In addition, this kinetic mechanism could enable catalysis by phospholipase C (PLC) to diminish PTEN activity (and thus encourage cell proliferation) by decreasing the concentration of PIP_2_. This mechanism could, for example, increase the invasiveness of prostate cancer cells having high levels of PLCγ [36].

Our continuous assay also enabled a detailed analysis of catalysis by PTEN-L. In addition to the striking 2.2-fold increase in *k*_cat_/*K*_M_ over wild-type PTEN at 0 mM NaCl, we find that PTEN-L does not respond to increased salt concentration in the same manner as does the wild-type enzyme. Salt concentration affects the value of *K*_M_ but does not introduce cooperativity.

**Figure 7:**
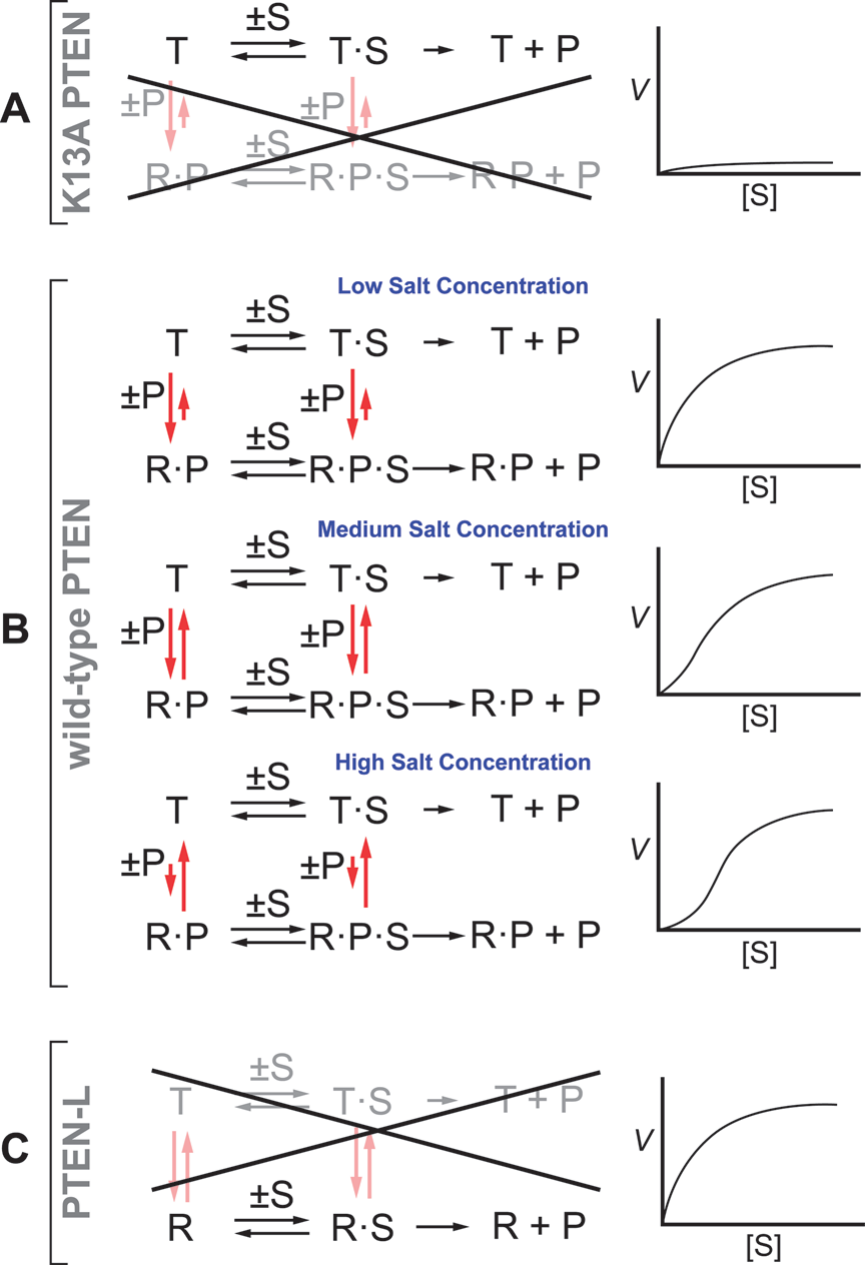
Notional schemes and diagrams for catalysis by K13A PTEN, wild-type PTEN, and PTEN-L. (A) The binding motif for PIP_2_ (P) is absent in K13A PTEN. As a result, the K13A variant remains in its tense (T) state for all substrate concentrations at any salt concentration. (B) Wild-type PTEN has complex kinetic behavior. Low salt concentration favors the binding of PIP_2_. The wild-type enzyme is in its relaxed (R) state and exhibits Michaelis–Menten kinetics. Increasing salt concentration leads to decreasing affinity for PIP_2_ and a greater fraction of the enzyme being in the T state. As more PIP_3_ (S) is hydrolyzed, the increasing concentration of P leads to a greater fraction of the enzyme being in the R state. (C) PTEN-L is in the R state at any salt concentration and is unaffected by P.

To summarize our findings, we put forth a model for catalysis by PTEN that is based on the canonical “tense” and “relaxed” states of hemoglobin [37]. In our model, PTEN can exist in “T” (low activity) and “R” (high activity) conformations (Fig. 7). The association of PIP_2_ with the N-terminal PIP_2_-binding site makes the R state more favorable. By removing the PIP_2_-binding site, we constrain K13A PTEN to the T state (Fig. 7A). In the T state, both *k*_cat_ and *K*_M_ have low values (Fig. 7B). For wild-type PTEN, the T state is retained until PIP_2_ is bound. At low salt concentration, the equilibrium constant for PIP_2_ association with the PIP_2_-binding site becomes high, and association is favorable, even at low concentrations of PIP_2_. Thus, the R state is more prevalent at low salt concentration and low substrate concentration. At high salt concentration, the binding of PIP_2_ is less favored because counterion-release provides less entropic gain, and the T state is prevalent at low substrate concentrations. As the reaction proceeds and the concentration of PIP_2_ increases, more PIP_2_ is bound by PTEN and more of the enzyme is present in the R state. Interestingly, our data indicate that PTEN-L is constrained to the R state and that increased concentrations of PIP_2_ do not effect any additional activation (Fig. 7C). Perhaps some of the additional N-terminal residues on PTEN-L bind to the PIP_2_ motif and mimic the binding of PIP_2_.

PTEN activity is eminently relevant to cancer [4]. Small changes in cellular PTEN activity are known to have large effects on tumor propagation [11], and exogenous PTEN-L shrinks tumors in mice [17]. Accordingly, small molecules that increase PTEN activity could have utility as cancer chemotherapeutic agents. In particular, molecules that encourage the conversion of endogenous PTEN from its T state to its R state could underlie a new chemotherapeutic strategy. Our continuous, colorimetric assay facilitates structure–function analyses, which could expedite the rational design of molecules that modulate PTEN activity. Moreover, our assay could be useful in high-throughput screens of molecular libraries for PTEN agonists.

### Conclusion

We have developed a continuous assay for catalysis by PTEN. Using that assay we affirm the presence of a product-binding site, uncover salt-dependence on that binding site, and assess activity by PTEN-L. In contrast to PTEN-L, which is always in a high activity state, wild-type PTEN toggles between a low activity and high activity state based on the concentration of salt and reaction product. These findings provide new information on catalysis by this highly important enzyme, and could empower workers seeking to develop PTEN-based chemotherapeutic agents.

## Materials and Methods

### Materials

*E. coli* BL21 (DE3) cells for protein purification were from Novagen (Madison, WI). Expression plasmid pET30B-PTEN was plasmid 20741 from Addgene (Cambridge, MA) and directs the expression of human PTEN with a C-terminal His_6_ tag. Expression plasmid pET30B-PTEN-K13A was derived using a Quikchange site-directed mutagenesis system from Agilent (Santa Clara, CA), and expression plasmid pET30B-PTEN-Long-S was derived by Gibson assembly [38] using a gBlocks gene fragment from Integrated DNA Technologies (Coralville, IA). In pET30B-PTEN-L-S, the CTG codon encoding residue 1 was changed to ATG, the signal sequence (codons 2–21) was removed, the ATG codon at the translation start site for wild-type PTEN was replaced with ATA (codon 174), and other codons in the appended upstream region were optimized for expression in *E. coli* without affecting the encoded protein sequence.

Terrific broth (TB) medium contained tryptone (12 g), yeast extract (24 g), K_2_HPO_4_ (72 mM), KH_2_PO_4_ (17 mM), and glycerol (4 mL). Columns of HisTrap HP, HiTrap Q HP, HiTrap Heparin HP, and Superdex G200 resins for protein purification were from GE Biosciences (Piscataway, NJ).

diC_8_-Phosphatidylinositol-3,4,5-trisphosphate (PIP_3_) was from Avanti Polar Lipids (Alabaster, AL) and Echelon Biosciences (Salt Lake City, UT). Bacterial nucleoside phosphorylase (PNPase) was product N2415 from Sigma–Aldrich (St. Louis, MO), dissolved in reaction buffer, and buffer-exchanged to remove residual phosphate. 7-Methyl-6-thioguanosine (MESG) was from Berry and Associates (Dexter, MI). Dithiobutylamine (DTBA [31]) was product 774405 from Sigma–Aldrich. All other chemicals and reagents were of commercial reagent grade or better, and were used without further purification.

### Production and Purification of PTEN

Methods for the expression and purification of PTEN were based on those of Ross and coworkers [20]. Briefly, a PTEN expression plasmid was transformed into *E. coli* strain BL21(DE3), and grown in TB supplemented with kanamycin (30 μM). Expression was induced at OD = 0.5–0.6 at 600 nm by the addition of isopropyl β-d-1-thiogalactopyranoside to 0.10 mM, and cells were grown for 20–22 h at 21°C to produce wild-type PTEN and its K13A variant, and 18°C to produce PTEN-L. Cells were harvested by centrifugation and lysed in 20 mM sodium phosphate buffer, pH 7.4, containing NaCl (0.50 M), imidazole (20 mM), and β-mercaptoethanol (0.7% v/v) with a French pressure cell. Lysate was clarified by centrifugation at 20,000*g*, and the soluble fraction was applied to a HisTrap HP column. Protein was eluted with 0.50 M imidazole, and fractions containing PTEN were purified further by chromatography on a HiLoad 26/60 G200 Superdex gel-filtration column. As a final step, wild-type PTEN and its K13A variant were purified by chromatography on a HiTrap Q anion-exchange column at pH 7.4, and PTEN-L was purified by chromatography on a HiTrap Heparin HP affinity column using an ÄKTA system from Amersham-Pharmacia (Piscataway, NJ), and the results were analyzed with the UNICORN Control System. Aliquots of protein were supplemented with DTT (10 mM), glycerol (25% v/v), and EDTA (2 mM), flash frozen in liquid nitrogen, and stored at −80°C. Protein concentrations were measured with a Bradford assay [39].

### Continuous Assay for the Enzymatic Activity of PTEN

We sought to establish the first continuous assay for the catalytic activity of PTEN. Our assay is based on the utilization of inorganic phosphate (P_i_) by PNPase, which catalyzes the reaction of P_i_ and 7-methyl-6-thioguanosine (MESG) to form ribose-1-phosphate and 7-methyl-6-thioguanine (Fig. 1B) [19]. This assay is continuous, taking advantage of the large absorbance difference of MESG and the free nucleobase, 7-methyl-6-thioguanine. The Δ*ε* for this reaction is 11 mM^−1^cm^−1^ at 360 nm.

The putative assay must be sensitive enough to respond to small amounts of released P_i_. Using the known value of Δ*ε*, we predicted that a release of 2 μM P_i_ would raise the absorbance in a 1-cm path-length cuvette by 0.022 OD. This absorbance increase can be measured readily with a typical spectrophotometer or plate reader. Also, PTEN requires a reducing environment for its catalytic activity as its active-site nucleophile is a cysteine thiolate. We found that PNPase is able to function in a highly reducing environment.

PNPase concentration was measured with a Bradford assay [39] and added to a concentration of 57 μg/mL (2 μM) in reaction buffer, which was 50 mM Tris-HCl buffer, pH 7.6, containing EDTA (2.0 mM), MESG (0.6 mM), and DTBA (40 mM). In our assays, we used the soluble diC_8_ version of phosphatidylinositol-(3,4,5)-trisphosphate as the substrate, as others have used in discontinuous assays [9,14–16,20]. Known concentrations of substrate (0–320 μM) were added to the buffer, and reactions were initiated by the addition of PTEN or PTEN-L to 20 nM, or K13A PTEN to 250 nM. As specified, some reactions contained NaCl (0, 100, or 200 mM) or another salt. Unlike in discontinuous assays [20], we did not add PIP_2_ product to the assay solution, so as to amplify any effects of PIP_2_ generated *in situ* on the reaction kinetics. Measurements of absorbance at 360 nM were recorded at 25°C with a Cary 60 UV–Vis spectrophotometer having a Varian Cary Single Cell Peltier temperature control accessory from Agilent Technologies (Santa Clara, CA). Data analysis was performed with Prism version 6 from Graphpad (San Diego, CA).
